# Fecal Microbiota Transplantation in Colorectal Cancer: From Immune Regulation to Immunotherapy

**DOI:** 10.3390/cancers18213426

**Published:** 2026-10-22

**Authors:** Xin Yang, Xiaoqin Ge, Shengping Gong, Ting Zhu, Chao Ni, Ruishuang Ma, Qingsong Tao

**Affiliations:** Department of Radiotherapy and Chemotherapy, The First Affiliated Hospital of Ningbo University, Ningbo 315211, China

**Keywords:** colorectal cancer, fecal microbiota transplantation, immunotherapy, short-chain fatty acids, tumor microenvironment

## Abstract

The gut microbiome influences colorectal cancer (CRC) progression and responses to immune checkpoint inhibitors (ICIs), with immunotherapy resistance remaining a major challenge in microsatellite-stable/mismatch repair-proficient (MSS/pMMR) CRC. This review examines the mechanisms and translational potential of fecal microbiota transplantation (FMT) in CRC immunotherapy, focusing on the interplay among microbial functions, metabolic alterations, and immune microenvironment remodeling. Current evidence suggests that FMT may influence antitumor immunity beyond restoring microbial diversity by transferring functionally relevant microbial communities, metabolic profiles, and immunomodulatory signals. These changes may affect antigen presentation, inflammatory signaling, T-cell activity, and myeloid-cell states, thereby shaping the tumor immune microenvironment and potentially modulating responses to PD-1/PD-L1 blockade. However, the clinical efficacy of FMT in CRC, the determinants of successful microbial engraftment, and the mechanisms linking donor-derived microbial functions to therapeutic outcomes remain incompletely understood. Integrating defined microbial consortia, metabolite-based interventions, and multi-omics-guided patient stratification may help advance FMT toward mechanism-driven precision microbiome therapy and inform strategies to improve CRC immunotherapy outcomes.

## Simple Summary

Colorectal cancer remains largely resistant to immunotherapy, particularly microsatellite-stable/mismatch repair-proficient tumors. The gut microbiota regulates antitumor immunity and treatment response. Fecal microbiota transplantation transfers donor-derived microbial communities and has emerged as a promising strategy to overcome immunotherapy resistance. This review summarizes how FMT enhances antigen presentation, rebalances inflammatory signaling, restores CD8^+^ T-cell function and plasticity, reprograms immunosuppressive myeloid cells, and rebuilds microbial metabolic networks involving short-chain fatty acids, bile acids, and tryptophan metabolites. These coordinated effects may convert immune-cold MSS/pMMR CRC into a more immune-responsive state. We also discuss context-dependent microbial functions and propose mechanism-driven, precision microbiome therapy.

## 1. Introduction

Colorectal cancer (CRC) is one of the most prevalent malignancies of the digestive system and remains a major global health burden. According to global cancer statistics for 2022, approximately 1.9 million new CRC cases and more than 900,000 CRC-related deaths occurred worldwide, making CRC the third most frequently diagnosed cancer and the second leading cause of cancer-related mortality [1]. CRC development and progression are driven by complex interactions among genetic predisposition, environmental exposures, dietary patterns, lifestyle factors, and host immune status. At the molecular level, CRC evolution involves dysregulation of canonical oncogenic pathways, including Wnt/β-catenin, KRAS, TP53, and transforming growth factor-β (TGF-β) signaling, together with epigenetic alterations such as DNA methylation, histone modifications, and non-coding RNA-mediated regulation. These molecular events contribute to adenoma–carcinoma progression, tumor heterogeneity, and therapeutic resistance [2,3,4,5].

Based on mismatch repair (MMR) status and microsatellite instability (MSI), CRC can be broadly classified into MSI-high/deficient mismatch repair (MSI-H/dMMR) and microsatellite-stable/proficient mismatch repair (MSS/pMMR) subtypes. MSI-H/dMMR tumors generally exhibit a high tumor mutational burden and increased neoantigen generation, facilitating antitumor immune recognition and contributing to their sensitivity to immune checkpoint inhibitors (ICIs), particularly programmed cell death protein 1 (PD-1)/programmed death-ligand 1 (PD-L1) blockade [6,7,8]. In contrast, approximately 85% of CRC cases are MSS/pMMR tumors, which are often characterized by limited effector T-cell infiltration, impaired antitumor immune activity, and an immunosuppressive tumor microenvironment (TME). These features contribute to their generally poor response to ICI monotherapy [9,10]. Therefore, converting MSS/pMMR CRC from an immune-refractory state into an immune-responsive state remains a major challenge in cancer immunotherapy.

In recent years, the gut microbiota has emerged as an important regulatory interface linking environmental exposures, inflammatory responses, metabolic processes, and antitumor immunity. Because CRC develops in a microbially enriched intestinal environment, tumor initiation and progression are influenced not only by intrinsic genetic alterations in tumor cells but also by interactions among intestinal microorganisms, epithelial barrier integrity, and tumor-associated microbial communities [11,12]. Several microorganisms have been implicated in CRC initiation and progression through distinct mechanisms. For example, *Fusobacterium nucleatum* (Fn) can promote tumor development through FadA-mediated epithelial adhesion, activation of Wnt/β-catenin signaling, and modulation of antitumor immune responses. Other studies have linked Fn to additional mechanisms of tumor-promoting activity, highlighting its multifaceted effects on CRC biology [13,14,15,16]. Meanwhile, pks-positive *Escherichia coli* produces colibactin, a genotoxic metabolite associated with DNA damage and genomic instability [17,18]. *Bacteroides fragilis* and *Peptostreptococcus anaerobius* have also been implicated in CRC progression through inflammatory activation and metabolic or immune remodeling [19,20]. Together, these findings indicate that the gut microbiota is not merely a passive bystander in colorectal tumorigenesis but an active participant in inflammation, tumor evolution, and potentially therapeutic resistance.

Conversely, microbial communities with immunomodulatory and barrier-protective properties may also influence CRC progression and treatment outcomes. Microorganisms such as *Akkermansia muciniphila*, *Bifidobacterium* species, and butyrate-producing bacteria have been associated with intestinal barrier maintenance, short-chain fatty acid (SCFA) production, and regulation of host immune responses [21,22,23]. However, microbial functions cannot be classified universally as either beneficial or harmful. Their biological effects may vary according to strain-specific characteristics, host immune status, disease stage, and ecological interactions within the microbial community. Microbial contributions to CRC should therefore be understood as dynamic ecological processes rather than a simple binary distinction between protective and pathogenic bacteria.

Beyond their effects on tumor initiation and progression, gut microorganisms can remodel the TME and influence responses to immunotherapy. Microbial signals can affect multiple immune cell populations, including CD8^+^ T cells, regulatory T cells (Tregs), myeloid-derived suppressor cells (MDSCs), tumor-associated macrophages (TAMs), and dendritic cells (DCs) [24,25,26]. Microbiota-derived metabolites, particularly SCFAs, secondary bile acids, and tryptophan metabolites, can regulate immune cell function through metabolic remodeling, epigenetic regulation, and receptor-mediated signaling [23,27,28]. In addition, microbial effects on intestinal barrier integrity, Toll-like receptor (TLR)-associated inflammatory signaling, antigen presentation, and myeloid cell polarization may contribute to changes in ICI responsiveness [21,29,30,31]. These interconnected mechanisms provide a rationale for investigating microbiome-based strategies to improve antitumor immunity.

Fecal microbiota transplantation (FMT) is a microbiome-based intervention in which a donor-derived microbial community is transferred to a recipient to restore or reshape the intestinal microbial ecosystem. FMT was initially established as an effective treatment for recurrent *Clostridioides difficile* infection. Its application in cancer research has expanded with increasing recognition of the interactions between the microbiota and host immunity [32,33]. Compared with observational studies that identify associations between microbial profiles and clinical outcomes, FMT provides an approach for investigating whether transferable microbial communities can influence host immune responses, metabolic profiles, and treatment sensitivity. Nevertheless, the biological effects of FMT depend on the transferred community, recipient characteristics, microbial engraftment, and treatment context.

The potential of FMT to modulate responses to cancer immunotherapy has been investigated clinically, initially in melanoma. Studies by Davar et al. and Baruch et al. reported that FMT from donors who responded to ICIs could restore or improve sensitivity to anti-PD-1 therapy in some patients with previously refractory melanoma [34,35]. Subsequent investigations have explored FMT in combination with ICIs in other solid tumors, providing further evidence that microbiome manipulation may influence immunotherapy outcomes [36,37]. However, results obtained in melanoma or mixed solid-tumor populations should not be directly interpreted as evidence of efficacy in CRC. Differences in tumor biology, baseline microbiota, treatment regimens, and study design must be considered when evaluating the clinical relevance of these findings.

In CRC, the application of FMT in combination with immunotherapy remains at an early stage of investigation. Preclinical studies suggest that transferring selected microbial communities may modify CRC-associated dysbiosis, alter inflammatory and metabolic pathways, and promote antitumor immune responses [38]. Clinical investigations have also begun to examine microbiome-based interventions in patients with CRC, including those with MSS disease, for whom effective immunotherapeutic options remain limited [39]. However, the available evidence remains preliminary, and the efficacy and reproducibility of FMT may be influenced by donor selection, microbial engraftment, recipient ecological conditions, safety considerations, and the accompanying treatment regimen. Moving from empirical transplantation toward mechanism-driven and precision microbiome-based interventions is therefore an important research priority [40,41,42].

In this review, we examine the interactions between the gut microbiota and antitumor immunity in CRC, focusing on the mechanisms through which FMT may influence immunotherapeutic responses. We discuss microbial regulation of antigen presentation and inflammatory signaling, changes in T-cell activity and the balance between effector and regulatory populations, myeloid cell reprogramming, and the immunomodulatory effects of microbiota-derived metabolites. We also assess the current translational evidence for FMT combined with ICIs, with particular attention to the challenges of treating MSS/pMMR CRC. By integrating mechanistic findings with emerging clinical evidence, this review aims to clarify the therapeutic potential and limitations of FMT and to identify priorities for developing more precise, reproducible, and mechanism-informed microbiome-based strategies for CRC immunotherapy.

## 2. Mechanisms by Which FMT Modulates Tumor Immunity

The gut microbiota does not influence immunotherapy in CRC through a single pathway. Instead, it reshapes the tumor immune microenvironment through dynamic interactions among microbial composition, metabolic function, and host immunity, ultimately determining the response to ICIs [43,44]. Increasing evidence suggests that the therapeutic value of FMT extends well beyond restoring microbial diversity or correcting dysbiosis. Rather, FMT transfers donor-derived functional microbial communities together with their metabolic capacity and immunoregulatory programs into the recipient, thereby simultaneously reconstructing the intestinal microbiota, rewiring microbial metabolism, and reprogramming host immunity [45,46]. Accordingly, FMT represents a systems-level microbiome intervention that orchestrates coordinated remodeling of the microbiota, microbial metabolism, and host immunity. Instead of targeting individual bacterial species, it restores the functional interactions between the microbiota and the host immune system, ultimately converting an immunosuppressive tumor microenvironment into one that is more permissive for antitumor immunity and more responsive to ICIs (Figure 1).

During CRC immunotherapy, FMT promotes the engraftment of beneficial microbial taxa, including *Faecalibacterium*, *Roseburia*, and *Akkermansia*, thereby restoring key microbial metabolic pathways responsible for the production of short-chain fatty acids (SCFAs), secondary bile acids, and tryptophan-derived metabolites [47,48]. These microbial metabolites subsequently drive immunometabolic reprogramming by inhibiting histone deacetylases (HDACs) and activating signaling pathways mediated by the aryl hydrocarbon receptor (AhR), Takeda G protein-coupled receptor 5 (TGR5), and farnesoid X receptor (FXR). Beyond metabolic regulation, FMT enhances dendritic cell (DC) maturation and antigen presentation, restores the cytotoxic activity of CD8^+^ T cells while maintaining the pool of TCF1^+^ progenitor exhausted T cells, and alleviates immunosuppression mediated by regulatory T cells and myeloid-derived suppressor cells. Collectively, these coordinated effects facilitate the conversion of microsatellite-stable/proficient mismatch repair (MSS/pMMR) tumors from an immune-cold to an immune-hot phenotype, thereby improving the efficacy of PD-1/PD-L1 blockade.

Taken together, the therapeutic benefits of FMT extend beyond changes in individual microbial taxa. Rather, they arise from coordinated remodeling of the gut microbiota, microbial metabolism, and host immunity, ultimately reshaping the tumor immune microenvironment. In the following sections, we discuss five interconnected mechanisms underlying these effects: enhancement of antigen presentation, inflammatory remodeling, restoration of T-cell function, myeloid immune remodeling, and microbiota-driven metabolic reprogramming.

### 2.1. FMT-Mediated Antigen Presentation

Antigen presentation represents the initiating step of adaptive antitumor immunity and serves as a fundamental determinant of response to ICIs. Tumor-derived antigens must first be captured and processed by antigen-presenting cells (APCs), followed by presentation through major histocompatibility complex (MHC) molecules to T cells, thereby inducing tumor-specific T-cell expansion and effector differentiation. However, in most MSS/pMMR CRC, impaired antigen recognition and immune priming limit effective immune activation. This is largely attributed to low tumor mutational burden, insufficient neoantigen generation, and defective antigen-presenting capacity within the tumor microenvironment, particularly reduced dendritic cell maturation and cross-presentation activity. Consequently, tumor antigens fail to efficiently initiate productive T-cell responses, contributing to the limited efficacy of PD-1/PD-L1 blockade in this immune-cold tumor subtype. Therefore, restoring antigen recognition and immune initiation represents an important strategy for converting MSS/pMMR CRC from an immune-refractory state toward a more immune-responsive phenotype [49,50].

Recent studies have expanded the role of the gut microbiota beyond regulation of downstream immune effector functions, demonstrating that microbial signals can influence early immune activation events by modulating APC differentiation, migration, and antigen-presenting capacity. As an intervention capable of transferring an entire microbial ecosystem, FMT provides a unique approach to investigate how microbiome alterations influence antigen presentation and subsequent immune activation. Unlike conventional microbiome profiling studies that primarily identify associations between microbial signatures and ICI responsiveness, FMT enables the functional transfer of donor-derived microbial communities and allows direct assessment of their ability to reshape APC activity and antitumor immunity in recipient hosts [51,52].

Mechanistically, intestinal microbes regulate APC function through microbial-associated molecular patterns (MAMPs). Microbial components, including lipopolysaccharide (LPS), peptidoglycan, and flagellin, can activate pattern recognition receptors (PRRs), such as Toll-like receptors (TLRs) and nucleotide-binding oligomerization domain (NOD)-like receptors, thereby promoting DC maturation and enhancing the expression of MHC-I/MHC-II molecules and costimulatory molecules, including CD80 and CD86. These changes ultimately improve tumor antigen presentation and facilitate T-cell activation. Uribe-Herranz et al. provided mechanistic evidence demonstrating that microbiota composition influences DC-mediated antigen cross-presentation and subsequently regulates CD8^+^ T-cell antitumor responses. Although this study was primarily performed in a radiotherapy-induced immune activation model, the identified microbiota–DC–T-cell regulatory axis provides important conceptual support for understanding how microbial remodeling may enhance immune sensitization in CRC [53].

Importantly, the immunological effects of the microbiota are unlikely to be determined solely by changes in bacterial abundance. Increasing evidence indicates that functional microbial configurations, rather than individual bacterial taxa, are more relevant for inducing effective immune programs. Tanoue et al. demonstrated that a defined consortium of commensal bacteria could promote tumor-specific CD8^+^ T-cell responses and enhance antitumor immunity. This study highlighted that immune-modulatory microbial communities can facilitate immune priming and improve tumor antigen recognition through coordinated functional interactions, rather than through expansion of a single bacterial species [29].

Beyond defined microbial consortia, several individual microbial members with immunomodulatory properties have also been implicated in regulating DC-mediated antigen presentation. Sivan et al. reported that *Bifidobacterium* enhanced DC-dependent antigen presentation, increased tumor-specific CD8^+^ T-cell activation, and improved responses to anti-PD-L1 therapy [22]. Similarly, Routy et al. demonstrated that enrichment of *Akkermansia muciniphila* was associated with improved response to PD-1 blockade, partly through enhanced IL-12-mediated immune activation and increased recruitment of antitumor T cells [21]. Together, these findings suggest that the immunological effects of FMT cannot be attributed to individual bacterial taxa alone; instead, FMT transfers a functional microbial ecosystem that collectively regulates dendritic cell maturation, antigen presentation, and downstream immune activation.

It is important to emphasize that FMT-mediated regulation of antigen presentation should not be interpreted as a simple enhancement of inflammatory activation. Instead, effective microbiome remodeling appears to restore a balanced interaction between microbial signals and host immunity, allowing APCs to achieve an appropriate activation state. Persistent and excessive microbial stimulation may trigger uncontrolled TLR/NF-κB signaling, leading to chronic inflammation and immune dysfunction. In contrast, functional microbial restoration may enhance antigen recognition and immune initiation while preventing pathological inflammatory activation.

Overall, the impact of FMT on antigen presentation reflects a broader process of immune ecosystem remodeling rather than a single microbial effect. Microbiota-derived signals and metabolites can regulate innate immune activation, promote DC maturation, and restore antigen cross-presentation capacity, thereby facilitating initial T-cell priming and subsequent antitumor responses. For immune-cold MSS/pMMR CRC, improving antigen presentation deficiency may represent one of the critical steps toward increasing sensitivity to ICIs.

### 2.2. FMT-Mediated Inflammatory Remodeling

Beyond regulating antigen presentation and immune priming, the gut microbiota continuously shapes the inflammatory landscape of CRC through interactions with host immune signaling networks. CRC develops within a highly microbial-rich intestinal environment, where microbial dysbiosis can lead to excessive accumulation of microbe-associated molecular patterns, persistent activation of pattern recognition receptors, and sustained production of pro-inflammatory cytokines, ultimately contributing to a chronic inflammatory state that favors immune suppression. Therefore, the immunomodulatory effects of FMT should not be interpreted simply as the enrichment of certain “beneficial bacteria.” Instead, FMT may restore immune homeostasis by re-establishing a balanced interaction between microbial signals and host inflammatory responses, thereby reducing pathological inflammatory stimulation while preserving immune activity required for antitumor responses [54,55,56].

During CRC initiation and progression, microbiota-driven inflammatory signaling represents a critical link between microbial imbalance and tumor immune escape. Among these pathways, Toll-like receptor (TLR)/NF-κB signaling is considered a central mediator of microbiota-induced inflammatory responses. Microbial-derived components, including lipopolysaccharide, lipoproteins, and other bacterial products, can activate host inflammatory programs through PRRs, resulting in sustained NF-κB activation and increased production of inflammatory cytokines such as IL-6, TNF-α, and IL-1β. Persistent low-grade microbial stimulation may reinforce this inflammatory state, further stabilizing the immune-refractory phenotype observed in MSS/pMMR CRC and creating a tumor microenvironment that is less permissive to immune checkpoint blockade [57,58]. Accordingly, the goal of FMT-mediated inflammatory regulation is unlikely to be complete suppression of inflammation; rather, it involves correcting excessive inflammatory inputs while restoring an appropriate inflammatory context that supports antitumor immunity.

In addition to the TLR/NF-κB pathway, the IL-23/IL-17 inflammatory axis represents another major mechanism through which intestinal microbes regulate CRC-associated inflammatory ecosystems. Grivennikov et al. demonstrated that microbiota-derived signals promote IL-23/IL-17-driven inflammatory responses and contribute to inflammation-associated colorectal tumorigenesis. Their findings indicate that microbial stimulation can reshape mucosal immune networks to sustain a tumor-promoting inflammatory environment, whereas microbial imbalance, barrier disruption, and abnormal immune activation may further amplify this process [59]. By restoring microbial community structure, improving intestinal barrier function, and reducing excessive microbial stimulation, FMT may therefore help restrain persistent inflammatory signaling and reshape the immune contexture of CRC.

Importantly, FMT-mediated regulation of inflammation should be viewed as immune inflammatory reprogramming rather than simple anti-inflammatory suppression. Effective antitumor immunity requires a certain degree of inflammatory signaling to facilitate immune cell recruitment, activation, and functional differentiation. However, prolonged microbial stimulation and uncontrolled activation of inflammatory pathways can instead promote immune dysfunction and therapeutic resistance. Mager et al. demonstrated that microbiota-derived signals regulate tumor immunity through the STING–type I interferon pathway, promoting immune activation programs and enhancing responses to immune checkpoint blockade. These findings suggest that microbiota-mediated immune modulation does not rely on nonspecific inflammatory amplification, but rather on resetting the threshold between immune activation and immune tolerance [26].

Overall, FMT-mediated inflammatory remodeling represents a dynamic process that rebalances microbial stimulation, host inflammatory responses, and immune effector functions within the tumor ecosystem. Unlike antigen presentation regulation, which primarily influences the initiation of immune responses, inflammatory remodeling determines whether activated immunity can be sustained within the tumor microenvironment without progressing toward immune exhaustion or tolerance. Therefore, restoration of inflammatory homeostasis represents an important mechanistic basis through which FMT may enhance immunotherapy responsiveness, particularly in immune-cold MSS/pMMR CRC.

### 2.3. FMT-Mediated T-Cell Functional Restoration

The efficacy of ICIs largely depends on the presence, activation status, and sustained cytotoxic activity of tumor-infiltrating T cells, particularly CD8^+^ cytotoxic T lymphocytes (CTLs). However, in most MSS/pMMR CRCs, persistent antigen exposure, metabolic constraints, and accumulated immunosuppressive signals within the tumor microenvironment gradually drive CD8^+^ T cells toward a dysfunctional exhausted state. This phenotype is characterized by sustained expression of inhibitory receptors, including PD-1, TIM-3, and LAG-3, together with reduced production of effector molecules such as IFN-γ, TNF-α, granzyme B, and perforin. Consequently, blockade of PD-1/PD-L1 signaling alone may be insufficient to fully restore T-cell antitumor activity. Reinvigorating exhausted T cells and restoring their functional plasticity therefore represents an important strategy for improving ICI responsiveness. Increasing evidence suggests that FMT influences T-cell immunity not simply by increasing T-cell recruitment or infiltration, but by reshaping T-cell functional states and promoting the transition from a hyporesponsive exhausted phenotype toward a more durable effector state [60,61]. For MSS/pMMR CRC, where chronic immunosuppressive pressure represents a major barrier to effective immunotherapy, restoring T-cell functional plasticity has become a key therapeutic consideration.

Recent work by Kang et al. provided mechanistic insights into how microbiota-derived functions regulate CD8^+^ T-cell activity in CRC. Through integrated analyses of CRC patient cohorts, multiple tumor models, and immune profiling approaches, the study demonstrated that the butyrate-producing bacterium *Roseburia intestinalis* was depleted in CRC patients, whereas supplementation with this bacterium markedly improved responses to anti-PD-1 therapy. Mechanistically, *R. intestinalis* colonization promoted the accumulation of Granzyme B^+^, IFN-γ^+^, and TNF-α^+^ CD8^+^ T cells within tumors, enhanced cytotoxic T-cell activity, and suppressed tumor growth. These findings provide direct evidence that specific functional microbial members can regulate CD8^+^ T-cell effector programs, suggesting that FMT may restore antitumor immunity by transferring microbial functional modules capable of supporting T-cell activation and persistence [62].

Beyond short-term T-cell activation, FMT may also influence long-term immune maintenance by shaping T-cell differentiation trajectories and memory formation. Fang et al. reported that microbiota-derived metabolic signals regulate CD8^+^ T-cell differentiation, memory development, and effector function, leading to enhanced IFN-γ production and improved tumor infiltration capacity. These findings suggest that microbiome-mediated regulation may promote durable antitumor immunity by altering the developmental fate and functional persistence of T cells, providing a potential explanation for the sustained immune benefits observed after microbiota-based interventions [63]. Consistently, Wong et al. summarized the complex interactions between gut microbiota and immune cells in CRC, highlighting that microbial signals can influence T-cell differentiation, functional states, and the overall tumor immune microenvironment, thereby affecting therapeutic responses [12].

Overall, the major contribution of FMT to T-cell immunity lies not in simply increasing the number of infiltrating T cells, but in restoring their functional competence within an immunosuppressive tumor ecosystem. Through recovery of immune-supportive microbial functions, modulation of T-cell differentiation programs, and alleviation of exhaustion-associated phenotypes, FMT may enhance the durability and cytotoxic capacity of CD8^+^ T cells, providing an important mechanistic basis for improving immunotherapy sensitivity in MSS/pMMR CRC.

### 2.4. FMT-Mediated Myeloid Immune Reprogramming

Myeloid cells represent more than simple immune effector populations during CRC immunotherapy; rather, they function as central regulators that shape the tumor immune ecosystem and influence therapeutic responsiveness. This role is particularly evident in MSS/pMMR CRC, where myeloid-derived suppressor cells, tumor-associated macrophages, and dysfunctional myeloid antigen-presenting cells frequently accumulate within the tumor microenvironment. Through multiple immunosuppressive mechanisms, including secretion of IL-10 and TGF-β, activation of ARG1 and iNOS pathways, and upregulation of PD-L1 expression, these myeloid populations restrict antitumor immunity and facilitate immune escape. Therefore, in addition to restoring T-cell activity, reversing myeloid-driven immunosuppression represents another important mechanism through which FMT may enhance sensitivity to ICIs [64,65].

Recent studies have highlighted the remarkable plasticity of myeloid cells, whose differentiation trajectories and functional states are continuously shaped by microbial composition, microbiota-derived signals, and metabolic inputs. By restructuring the intestinal microbial ecosystem, FMT has the potential to alter microbial inflammatory cues and metabolite availability, thereby influencing myeloid cell recruitment, differentiation, and functional polarization. In this context, myeloid cells may serve as a critical interface linking microbiome remodeling with tumor immune responses [66].

One major aspect of FMT-mediated myeloid regulation involves the remodeling of MDSC-associated immunosuppressive networks. MDSCs are key contributors to the immune-excluded phenotype observed in MSS/pMMR CRC and suppress antitumor immunity through multiple mechanisms, including depletion of extracellular arginine, production of reactive oxygen species (ROS) and nitric oxide, enhanced PD-L1 expression, and expansion of regulatory immune populations [64,67]. Microbial dysbiosis can promote MDSC recruitment and maintain their suppressive phenotype through persistent inflammatory stimulation and abnormal metabolic signaling. Conversely, FMT-mediated restoration of microbial balance may reduce immunosuppressive signaling inputs and alleviate myeloid-mediated immune suppression.

Microbiota-derived metabolites represent important regulators of MDSC biology. Short-chain fatty acids, secondary bile acid derivatives, and tryptophan metabolites not only participate in immune cell metabolic regulation but also influence myeloid cell differentiation and transcriptional programs through specific signaling pathways. For example, SCFAs can modulate inflammatory phenotypes and metabolic states of myeloid cells, whereas dysregulated microbial metabolism may generate signals that reinforce immunosuppressive myeloid programs [47,68].

Evidence from CRC models further supports the close relationship between microbial imbalance and myeloid immune dysfunction. Hu et al. demonstrated that alterations in the intestinal fungal community promoted the accumulation of immunosuppressive myeloid populations and contributed to CRC immune escape. Modulating abnormal fungal-derived signals reduced immunosuppressive cell infiltration and restored antitumor immune activity, highlighting the broader role of microbial ecosystems beyond bacterial composition alone [69]. In addition, studies combining ZQFZ with PD-1 blockade showed that increasing SCFA production and activating GPR109A-associated signaling reduced MDSC abundance and promoted macrophage transition toward antitumor phenotypes, suggesting that microbiota-derived metabolic regulation may contribute to enhanced responsiveness to ICIs [70].

Beyond MDSCs, TAMs represent another major target of microbiota-mediated immune regulation. TAM populations in CRC exhibit substantial functional heterogeneity. Immunosuppressive M2-like TAMs are frequently enriched within tumors and promote immune escape through enhanced angiogenesis, extracellular matrix remodeling, immune exclusion, and increased PD-L1 expression, whereas M1-like macrophages with inflammatory characteristics contribute to antitumor immune surveillance [71].

FMT may influence macrophage functional states by reshaping microbial-derived signals and metabolic environments. On one hand, microbial metabolites can regulate macrophage metabolic pathways and inflammatory transcriptional programs, facilitating their transition toward immune-active states. On the other hand, dysbiotic microbial communities may maintain immunosuppressive TAM phenotypes through persistent inflammatory signaling. Studies investigating microbiota–macrophage interactions have demonstrated that microbial ecosystems contribute to macrophage homeostasis, inflammatory regulation, and tissue repair processes, emphasizing the importance of microbial context in determining macrophage function [72].

Overall, FMT-mediated myeloid immune remodeling represents an important mechanism underlying microbiome-associated enhancement of CRC immunotherapy. Unlike direct restoration of T-cell effector function, myeloid regulation primarily targets upstream immune barriers that contribute to ICI resistance. By modulating MDSC accumulation, TAM polarization, and microbiota-derived inflammatory and metabolic signals, FMT may reshape the tumor immune environment from an immunosuppressive state toward a more permissive immune context. Consequently, myeloid immune characteristics may serve as potential biomarkers for evaluating FMT efficacy, predicting ICI responsiveness, and identifying patients most likely to benefit from precision microbiome-based interventions.

### 2.5. FMT-Mediated Metabolic Remodeling

Beyond changes in microbial composition and immune cell regulation, another important mechanism underlying FMT-mediated immunomodulation involves the restoration and reconstruction of microbial metabolic networks. Increasing evidence indicates that the biological effects of FMT cannot be explained simply by the expansion of specific bacterial taxa. Instead, FMT transfers donor-derived functional metabolic modules that reshape the metabolic environment experienced by host immune cells, thereby influencing immune responsiveness in CRC. Microbiota-derived metabolites, including SCFAs, tryptophan metabolites, secondary bile acids, and microbial-derived nucleoside metabolites, act as important signaling mediators connecting intestinal ecosystems with antitumor immunity. Through epigenetic regulation, immune metabolic reprogramming, and receptor-dependent signaling pathways, these metabolites can influence immune cell function and determine the outcome of ICI therapy [73,74]. Therefore, unlike FMT-mediated regulation of antigen presentation, T-cell activation, or myeloid immune states, metabolic remodeling emphasizes how microbial functional outputs reshape host immunity and the tumor ecosystem through biochemical signaling.

Among microbiota-derived metabolites, SCFAs, particularly butyrate, have emerged as key functional mediators of FMT-associated immune regulation. SCFAs are primarily produced by fiber-fermenting bacteria, including *Faecalibacterium*, *Roseburia*, and members of the *Ruminococcaceae* family. Beyond maintaining intestinal barrier integrity, these metabolites function as signaling molecules that directly regulate immune cell activity. Luu et al. systematically investigated the role of SCFAs in antitumor immunity and demonstrated that microbiota-derived butyrate and valerate enhanced CTL function by inhibiting class I histone deacetylase (HDAC) activity and promoting mTOR-associated metabolic programs. These metabolic changes increased the expression of antitumor effector molecules, including IFN-γ and TNF-α, thereby strengthening T-cell-mediated immune responses [75]. This study suggests that the immunological effects of FMT may depend less on restoring microbial composition alone, but rather on re-establishing the metabolic capacity of donor-derived microbial communities to continuously generate immune-regulatory signals.

In the context of CRC immunotherapy, increasing attention has been directed toward the role of microbial metabolites in maintaining T-cell function and modulating ICI responsiveness. He et al. reported that microbiota-derived butyrate enhanced CD8^+^ T-cell antitumor activity through an ID2-dependent IL-12 signaling pathway, thereby improving immune-mediated tumor control [76]. This finding indicates that butyrate is not merely an energy substrate but also functions as an immune-regulatory molecule capable of promoting CD8^+^ T-cell effector programs, enhancing IFN-γ production, and strengthening cytotoxic responses. Furthermore, alterations in microbial metabolic activity may influence the local immune environment within tumors, as microbiota-derived metabolic signals have been implicated in regulating T-cell persistence and exhaustion states. These findings suggest that metabolic remodeling represents an important determinant of responsiveness to ICIs [77].

The immunological effects of FMT-associated metabolic remodeling extend beyond SCFAs and involve a broad spectrum of bioactive microbial metabolites. For example, inosine produced by *Bifidobacterium pseudolongum* can activate the adenosine A2A receptor on T cells in the presence of appropriate costimulatory signals, promoting Th1-associated immune responses and enhancing the efficacy of immune checkpoint blockade [78]. In addition, microbial-derived tryptophan metabolites regulate T-cell differentiation, IL-22 production, and mucosal immune homeostasis through aryl hydrocarbon receptor (AhR) signaling. Secondary bile acid metabolites can also influence immune cell function and immune tolerance through receptors such as farnesoid X receptor (FXR) and Takeda G protein-coupled bile acid receptor 1 (TGR5) [79,80]. Collectively, these findings indicate that FMT-mediated immune regulation does not rely on a single metabolite but rather involves coordinated restoration of multiple microbial metabolic pathways that collectively shape tumor immunity.

In CRC, FMT-mediated metabolic remodeling involves not only restoration of beneficial metabolites such as SCFAs but also broader reprogramming of immune metabolism within the tumor microenvironment. Tumors are characterized by nutrient competition, hypoxic conditions, and metabolic stress, all of which restrict effector T-cell function and promote immune dysfunction. By restoring SCFA-producing microbial communities and rebuilding microbial metabolic networks, FMT may improve the availability of immune-supportive metabolites and alleviate immunosuppressive metabolic constraints within tumors [24,81]. Therefore, the central value of FMT lies not simply in altering “which microbes are present,” but in restoring the functional metabolic outputs encoded by microbial communities and rebuilding a metabolic environment favorable for antitumor immunity.

Importantly, metabolic remodeling mediated by FMT should not be viewed as a strategy aimed at increasing the abundance of individual bacterial species. Rather, the goal is to recover microbial ecological functions capable of regulating immune responses. Recent studies have shown that dietary fiber interventions can expand butyrate-producing bacteria and enhance anti-PD-1 therapeutic efficacy, suggesting that the beneficial effects associated with FMT may arise from transferable metabolic functions rather than the complete transfer of an entire microbial community [82]. These findings support a transition from conventional “microbial replacement” approaches toward “functional metabolic supplementation,” including defined microbial consortia, postbiotics, and targeted metabolite combinations, to improve ICI responsiveness.

Overall, FMT-mediated metabolic remodeling represents a critical bridge connecting microbial alterations with antitumor immune responses. Through the regulation of SCFAs, tryptophan metabolites, secondary bile acids, and other microbial-derived functional molecules, FMT can influence T-cell activity, immune metabolic balance, and the overall tumor immune ecosystem. Accumulating evidence from microbiota- and FMT-based studies supports these immunometabolic interactions in CRC immunotherapy, as summarized in Table 1. Future precision microbiome-based therapies in CRC are therefore unlikely to focus solely on identifying optimal donors; instead, efforts will increasingly aim to define and reproduce immune-enhancing metabolic networks through engineered microbial consortia, metabolite-based interventions, and next-generation microbiome therapeutics.

## 3. Functional Plasticity of Tumor-Associated Microbiota

### 3.1. Tumor-Promoting Effects and Immune Escape Mediated by Fusobacterium nucleatum

Fn represents one of the most extensively studied tumor-associated microorganisms in CRC and exhibits a complex dual function in tumor biology, contributing not only to tumor initiation and progression but also to modulation of host immune responses. Multiple clinical studies have demonstrated that Fn is enriched in CRC tissues and is associated with tumor development, invasive behavior, therapeutic resistance, and unfavorable clinical outcomes [80,88]. Unlike conventional commensal microorganisms, Fn possesses strong adhesive, invasive, and immunomodulatory capabilities, allowing direct interaction with intestinal epithelial cells and regulation of inflammatory signaling networks and immune microenvironments. Therefore, Fn has been considered an important ecological mediator linking microbial colonization, aberrant tumor signaling activation, and immune escape (Table 2).

During the early stages of colorectal tumorigenesis, Fn contributes to malignant transformation primarily through promotion of epithelial proliferation and inflammatory activation. Rubinstein et al. demonstrated that the Fn surface adhesin FadA binds to E-cadherin on epithelial cells, leading to β-catenin release and activation of the Wnt/β-catenin signaling pathway. This interaction also induces the expression of inflammatory genes, thereby promoting intestinal epithelial proliferation and tumorigenic transformation [83]. Subsequent studies further revealed that Fn enhances CRC cell proliferation, migration, and invasion through induction of Annexin A1 expression and amplification of Wnt/β-catenin signaling activity, further supporting its role as a microbial driver of CRC progression [89]. Beyond promoting intrinsic malignant properties of tumor cells, Fn can facilitate immune escape through multiple mechanisms. Gur et al. reported that the Fn surface protein Fap2 interacts with the inhibitory immune receptor TIGIT, suppressing NK-cell and T-cell activation and reducing immune-mediated tumor recognition and elimination [84]. In addition, Fn infection can induce immune checkpoint expression in tumor cells. Gao et al. demonstrated that Fn promotes PD-L1 expression in CRC cells and enhances tumor immune evasion, suggesting that Fn directly contributes to the establishment of an immunosuppressive tumor state [90].

Accumulating evidence indicates that Fn does not act exclusively through tumor cells but also reshapes the immune microenvironment by regulating immune cell function. Fn-induced microbial stimulation promotes inflammatory cytokine production and affects tumor-associated macrophage (TAM) phenotypes. Through TLR4/NF-κB-associated inflammatory signaling, Fn can facilitate the acquisition of immunosuppressive macrophage characteristics, reinforce an immune-suppressive microenvironment, and promote CRC progression [85]. Moreover, Fn enrichment has been associated with reduced CD8^+^ T-cell activity, increased infiltration of immunosuppressive populations, and abnormal inflammatory activation within tumors, further highlighting its capacity to regulate antitumor immunity at multiple levels [86].

Collectively, the role of Fn in CRC cannot be explained by a single tumor-promoting mechanism. Instead, Fn contributes to colorectal tumorigenesis and immune escape through coordinated regulation of epithelial signaling, inflammatory networks, immune checkpoint pathways, and the tumor immune microenvironment. However, the tumor-promoting properties of Fn appear to be strongly dependent on ecological and therapeutic contexts rather than representing an absolute biological feature. Recent studies have revealed that under specific immunotherapy conditions, Fn may paradoxically enhance PD-1 blockade responsiveness through metabolite-mediated immune remodeling. This functional duality provides an important conceptual basis for understanding how traditionally tumor-associated microbes may contribute to antitumor immunity under selected conditions [92].

### 3.2. Immunotherapy Sensitization by Traditionally Tumor-Promoting Microbes

Historically, CRC-associated microorganisms have been broadly categorized as either “tumor-promoting” or “tumor-suppressive” based on their reported associations with tumor progression and host immune regulation. However, accumulating evidence from cancer microbiome research suggests that this binary classification is insufficient to capture the functional complexity of microbial ecosystems. Microbial effects are not solely determined by taxonomic identity but are shaped by multiple factors, including strain-specific characteristics, host immune status, spatial localization, metabolic activity, and therapeutic pressure. Consequently, the same microorganism may exert distinct, or even opposing, biological effects under different ecological conditions and disease stages. Understanding microbiota–tumor interactions therefore requires moving beyond microbial taxonomy toward a functional and ecological perspective [93].

This concept has become particularly relevant in the context of cancer immunotherapy. Earlier studies mainly focused on tumor-associated microbes as drivers of CRC progression through inflammatory activation, oncogenic signaling, and immune suppression. However, recent findings indicate that certain microorganisms do not function simply as promoters or inhibitors of tumor growth; instead, they influence therapeutic outcomes through their functional genes, metabolic products, and interactions with host immune networks. During ICI therapy, microbiota-derived signals can regulate innate immune activation, antigen presentation, and immune cell functional states, thereby reshaping the tumor immune ecosystem and influencing treatment responsiveness [94].

The emerging evidence surrounding Fn provides a compelling example of this functional complexity. Although Fn has traditionally been regarded as a pro-tumor microorganism in CRC, recent studies have revealed that its biological effects may vary substantially depending on the therapeutic context. Wang et al. demonstrated that Fn could enhance immunotherapy sensitivity in MSS CRC. Through transplantation of Fn-high patient-derived fecal microbiota into germ-free MSS CRC mouse models, the study showed that Fn-enriched microbiota improved responses to anti-PD-1 therapy. Mechanistically, Fn-derived butyrate inhibited HDAC3/8 activity in CD8^+^ T cells, promoted H3K27 acetylation at the *Tbx21* promoter region, increased TBX21 expression, reduced PD-1 expression, and alleviated CD8^+^ T-cell exhaustion, ultimately enhancing anti-PD-1 efficacy [39]. These findings challenge the conventional view of Fn as an exclusively tumor-promoting bacterium and demonstrate that microbial functions are strongly influenced by tumor subtype, ecological niche, and metabolic output.

Beyond Fn, other microorganisms traditionally associated with tumor development have also demonstrated context-dependent immunomodulatory properties. Certain *Bacteroides* species, for example, can influence immunotherapy responses by regulating dendritic cell activation, T-cell recruitment, and cytokine production. Similarly, microbial-derived metabolites can act as immune-regulatory mediators that shape antitumor immune responses. These observations suggest that microbiota-mediated therapeutic effects cannot be attributed to the presence or absence of individual microbial species alone, but rather depend on the integrated functional capacity of microbial communities and their metabolic interactions with the host.

In addition to tumor-associated microorganisms, functional members of healthy microbial ecosystems have also shown potential to enhance antitumor immunity. Weis et al. reported that a capsular polysaccharide produced by *Bacteroides uniformis* isolated from the healthy human microbiota activated a Lag-3-associated NK-cell immune axis, enhanced immune surveillance in colon cancer, and improved responses to immunotherapy. This study further emphasized that microbiome-based interventions should not simply aim to increase the abundance of “beneficial bacteria”; instead, the critical objective is to identify transferable microbial functions and molecular components capable of driving antitumor immune responses [87].

Taken together, the phenomenon of “tumor-promoting microbes enhancing immunotherapy” does not contradict their established roles in carcinogenesis. Rather, it highlights the remarkable functional plasticity of the tumor microbiome. Under chronic inflammatory conditions and natural disease progression, microorganisms such as Fn may promote CRC development through epithelial transformation, persistent inflammatory activation, and immune suppression. However, under specific therapeutic conditions, microbial metabolites, microbe-associated molecular patterns (MAMPs), and ecological interactions among microbial communities may be redirected toward immune activation and treatment sensitization. Therefore, future microbiome research should move beyond measuring microbial abundance and composition, and instead focus on strain-level functional differences, spatial ecological organization, and metabolic outputs to advance the transition from microbiome taxonomy toward functional microbiome ecology [93].

### 3.3. Ecological Context Dependency of Microbial Functions

From an ecological perspective, the dual biological roles exhibited by Fn reflect a broader principle of functional context dependency within the tumor microbiome. Microorganisms do not possess inherently fixed “beneficial” or “harmful” properties; instead, their biological outputs are shaped by the surrounding ecological environment, including host immune status, tumor metabolic conditions, spatial localization, microbial interactions, and therapeutic pressures. The same microorganism may activate distinct molecular pathways and generate different biological consequences depending on the ecological niche in which it resides. Therefore, microbiota–tumor interactions should not be interpreted through a simple classification of tumor-promoting versus tumor-suppressive microbes, but rather as dynamic ecological relationships that continuously evolve with disease progression and therapeutic intervention [95,96].

The ecological dependence of microbial function is particularly evident in CRC-associated microorganisms. For example, *Bacteroides fragilis* has traditionally been considered a tumor-promoting microorganism due to its ability to produce *B. fragilis* toxin, induce inflammatory signaling, and contribute to colorectal tumorigenesis. However, the immunological consequences of *B. fragilis* colonization may vary depending on host genetic background, local inflammatory conditions, and the composition of surrounding microbial communities [91]. Similarly, *Peptostreptococcus anaerobius* has been implicated in CRC development through regulation of host metabolic pathways and inflammatory signaling, but its biological effects are also influenced by ecological interactions with other microbial populations and the specific tumor environment [20].

These findings collectively indicate that CRC-associated microorganisms cannot be assigned to a single functional category based solely on their taxonomic identity. Instead, their ultimate biological effects emerge from the interaction between microbial traits, ecological context, metabolic activity, and host immune regulation. In this regard, the tumor microbiome should be viewed as a dynamic functional ecosystem rather than a fixed collection of individual microbial species.

This ecological perspective also has important implications for microbiome-based therapeutic strategies. Traditional approaches have largely focused on identifying specific “harmful” microbes for elimination or “beneficial” microbes for enrichment. However, accumulating evidence suggests that therapeutic outcomes may depend less on the presence or absence of individual taxa and more on whether microbial communities possess specific functional capacities capable of regulating host immunity. Therefore, future microbiome interventions should prioritize the identification of transferable microbial functions, including strain-level characteristics, spatial distribution patterns, and metabolic networks, rather than relying solely on taxonomic classification.

Such a shift from microbiome taxonomy toward functional ecology provides a more comprehensive framework for understanding microbial contributions to CRC progression and immunotherapy response. By defining the ecological conditions that determine microbial behavior, future precision microbiome therapies may move beyond conventional microbial replacement strategies toward targeted modulation of functional microbial networks, enabling more predictable and personalized approaches for enhancing cancer immunotherapy efficacy [97].

## 4. Future Perspectives: From FMT to Precision Microbiome-Based Therapy

Although FMT has demonstrated substantial mechanistic potential in enhancing CRC immunotherapy, its clinical translation remains constrained by several unresolved challenges. Current evidence supporting the combination of FMT and immunotherapy is largely derived from preclinical models, mechanistic studies, and small-scale early-phase clinical trials, whereas robust clinical data specifically in CRC remain limited. In other tumor types, particularly melanoma, FMT from ICI-responsive donors has shown encouraging clinical signals by partially restoring anti-PD-1 sensitivity in previously refractory patients [35,36]. However, clinical studies evaluating FMT-based interventions in CRC remain relatively small and heterogeneous, limiting the ability to establish reliable conclusions regarding molecular subtype-specific efficacy. For example, the exploratory ChiCTR2100046768 trial investigated FMT combined with tislelizumab and fruquintinib in patients with refractory MSS metastatic CRC. Although preliminary disease control and immune modulation signals were observed, the open-label, single-arm design and limited sample size prevent definitive assessment of therapeutic efficacy or identification of responsive patient populations [98]. Consistent with recent systematic analyses, FMT represents a promising strategy for enhancing cancer treatment responses, but its clinical value requires validation through larger, well-controlled, and mechanistically designed trials. Future studies should evaluate FMT-based combinations with PD-1/PD-L1 blockade, CTLA-4 inhibition, radiotherapy, chemotherapy, or antiangiogenic therapies through multicenter randomized clinical investigations.

A major challenge for clinical implementation is the standardization of donor selection. Current FMT studies generally classify donors as either “healthy donors” or “ICI responder donors”; however, these broad categories do not necessarily capture the functional microbial characteristics responsible for therapeutic sensitivity. Emerging evidence suggests that FMT efficacy is more closely associated with transferable microbial functions, including specific signaling pathways, metabolic capacities, strain-level engraftment potential, and metabolite profiles, rather than donor identity itself [99]. Strain-tracking studies have further demonstrated that microbial engraftment after FMT is influenced by interactions between donor-derived strains, recipient baseline microbiota composition, and ecological competition within the gut environment. Therefore, simply confirming donor origin is insufficient to explain clinical outcomes [100]. Future approaches should establish function-oriented donor repositories by integrating 16S rRNA sequencing, metagenomics, metabolomics, and immunological profiling, together with standardized biomarkers for donor evaluation and strain-level monitoring, to improve the reproducibility and controllability of FMT-based interventions.

Beyond donor-related factors, intrinsic heterogeneity among CRC patients represents another major barrier to successful translation. CRC patients differ substantially in molecular subtype, primary versus metastatic tumor ecology, prior antibiotic exposure, age-associated immune alterations, and bacterial–fungal community structures, all of which may influence microbial engraftment and subsequent immune remodeling. Moreover, intestinal microbiota and intratumoral microbial communities may form interconnected ecological networks, meaning that fecal microbiome profiling alone may not fully reflect the immune landscape within tumors. Future clinical strategies should therefore incorporate recipient ecological context and integrate multidimensional immune and microbiome biomarkers, including tumor-infiltrating lymphocyte density, CD8^+^/Treg ratios, SCFA levels, myeloid cell infiltration patterns, strain engraftment efficiency, and microbial diversity metrics, to enable more precise patient stratification and improve interpretation of clinical outcomes [101].

Safety and regulatory considerations remain additional obstacles for the widespread application of FMT in oncology. Although microbiome-based therapeutics have achieved regulatory milestones in recurrent *Clostridioides difficile* infection, including FDA-approved products such as Rebyota and Vowst, cancer patients receiving immunotherapy represent a distinct population requiring careful evaluation of potential risks. These concerns include pathogen transmission, dissemination of antimicrobial resistance genes, and unpredictable inflammatory complications. Previous reports of antibiotic-resistant bacterial transmission following FMT highlight the importance of stringent donor screening and comprehensive safety monitoring, particularly in immunocompromised patients undergoing anticancer treatment [40]. Systematic reviews have further emphasized that although FMT-associated adverse events are generally manageable, the complexity of microbial ecosystems, donor variability, and recipient vulnerability necessitate standardized, traceable, and regulated procedures before broad clinical adoption [102]. Therefore, transitioning from conventional whole-stool transplantation toward defined microbial consortia, targeted metabolite replacement, engineered microorganisms, or “deconstructed FMT” approaches may provide a more controllable and regulatory-compatible strategy.

Based on these challenges, future clinical development of microbiome-based therapies in CRC will likely require a stepwise framework. First, patient-derived FMT models, experimental tumor systems, and multi-omics approaches should be integrated to identify microbial signatures, metabolic pathways, and immune phenotypes associated with ICI responsiveness. Second, functional validation should be performed using individual strains, defined microbial consortia, key metabolites, or dietary interventions such as fiber supplementation to determine their causal roles in immune regulation and tumor control. Finally, rigorously stratified clinical trials in MSS/pMMR CRC populations should evaluate the safety and efficacy of microbiome-based strategies combined with PD-1/PD-L1 blockade, antiangiogenic therapy, radiotherapy, or chemotherapy. Current preclinical and early clinical studies suggest that microbiome-enhancing approaches have considerable therapeutic potential and can influence microbial engraftment and immune activation; however, definitive clinical benefits require larger trials with clearly defined mechanistic endpoints [103].

Overall, precision microbiome-based therapy represents a promising direction for improving immunotherapy outcomes in CRC, but successful clinical translation will require overcoming four major challenges: insufficient clinical evidence, donor standardization, patient heterogeneity, and safety regulation. Through mechanism-driven microbial selection, functional replacement strategies, and biomarker-guided clinical validation, future microbiome interventions may provide individualized therapeutic opportunities for patients with MSS/pMMR CRC. These efforts will ultimately facilitate the transition of microbiome-based approaches from exploratory concepts toward standardized, reproducible, and clinically applicable precision therapies [104].

## 5. Conclusions

The gut microbiota is an active modulator of CRC immunity and immunotherapy responsiveness, extending its role beyond tumor development to the modulation of host immune states and therapeutic outcomes. Although ICIs have demonstrated substantial clinical benefits in MSI-H/dMMR CRC, resistance remains a major challenge in MSS/pMMR disease. Current evidence highlights the potential of FMT to influence antitumor immunity through interconnected mechanisms involving antigen presentation, inflammatory signaling, T-cell function, myeloid-cell states, and microbiota-derived metabolites. These findings suggest that the immunological effects of FMT cannot be explained solely by changes in microbial diversity or the abundance of individual bacterial taxa but instead reflect the combined functional activities of microbial communities and their interactions with the host immune system.

An important insight emerging from this review is that microbial effects on CRC immunotherapy are context-dependent rather than strictly determined by bacterial taxonomy. The dual roles of microorganisms such as Fnillustrate how the same microbial species may contribute to tumor progression in one setting while influencing therapeutic immune responses through distinct metabolic or immunomodulatory mechanisms in another. Therefore, understanding microbiota-mediated immunotherapy responses requires consideration of microbial function, metabolic activity, and the surrounding immune context. Overall, FMT provides a valuable framework for understanding the relationship between microbial ecosystems and antitumor immunity, while its clinical efficacy in CRC, particularly in MSS/pMMR tumors, remains to be established through rigorous clinical evidence.

## Figures and Tables

**Figure 1:**
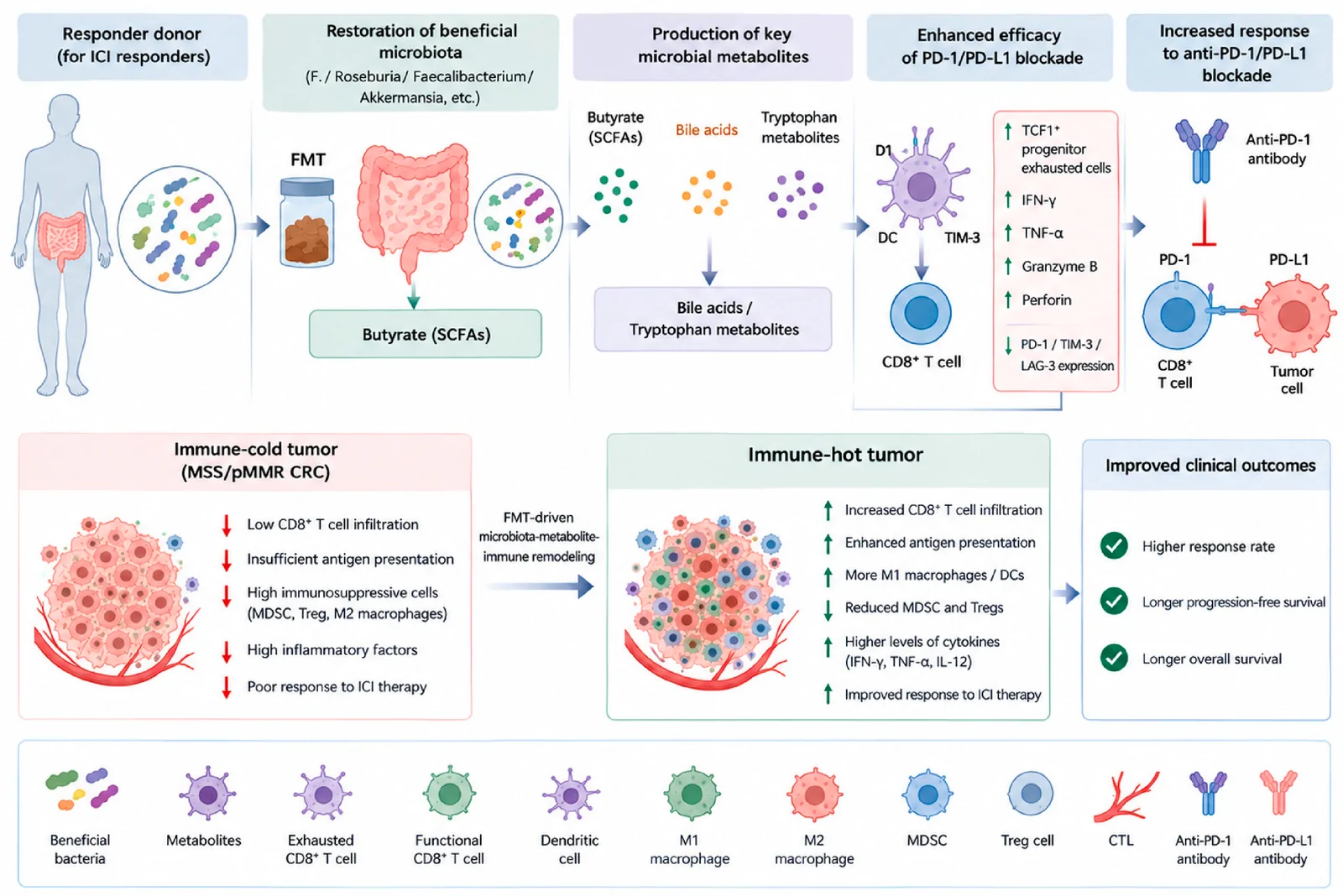
Proposed mechanisms by which fecal microbiota transplantation (FMT) modulates the tumor immune microenvironment and enhances the efficacy of immune checkpoint inhibitors in colorectal cancer. The figure illustrates how FMT-mediated remodeling of the gut microbiota and microbial metabolism may regulate dendritic cell maturation and antigen presentation, inflammatory cytokine networks, the balance between CD8^+^ T cells and regulatory T cells (Tregs), and myeloid-cell populations. It also highlights the potential roles of short-chain fatty acids, secondary bile acids, and tryptophan-derived metabolites in immunometabolic regulation. Together, these interconnected pathways may alleviate local immunosuppression and promote antitumor immune responses, potentially improving sensitivity to PD-1/PD-L1 blockade, particularly in microsatellite-stable/proficient mismatch repair (MSS/pMMR) colorectal cancer.

**Table 1:** Representative studies demonstrating microbiota- and FMT-mediated immune remodeling in colorectal cancer immunotherapy.

Mechanistic Category	Microbial Factor/Intervention	Molecular Mechanism	Immune Consequence	Implication for FMT-Based CRC Immunotherapy	Representative Study
Antigen presentation and immune priming	*Bifidobacterium* spp.	Commensal *Bifidobacterium* enhances dendritic cell activation and tumor antigen presentation	Increased tumor-specific CD8^+^ T-cell priming and improved anti-PD-L1 efficacy	Supports the concept that FMT may transfer immune-activating microbial functions that enhance antigen presentation	Ref. [22]
	Gut microbiota composition	Microbiota regulates dendritic cell-mediated antigen cross-presentation	Enhanced CD8^+^ T-cell activation after immune stimulation	Provides mechanistic evidence linking microbial remodeling with immune initiation	Ref. [53]
	Microbiota-derived immune signals	Microbial signals promote dendritic cell activation	Increased antitumor immune responses	Suggests FMT may restore APC function through microbial immune signals	Ref. [52]
	Defined microbial consortium	Selected commensal bacteria induce tumor-reactive CD8^+^ T-cell responses	Enhanced systemic antitumor immunity	Supports development of defined microbial consortia as alternatives to conventional FMT	Ref. [29]
Inflammatory remodeling and immune homeostasis	Gut microbiota dysbiosis	FMT reshapes microbiota and inflammatory pathways	Improved immune activation and tumor control	Directly supports FMT-mediated inflammatory remodeling in CRC	Ref. [54]
	Microbial products	Microbial products activate IL-23/IL-17 inflammatory axis	Promotes inflammatory tumor microenvironment	Demonstrates the importance of microbiota-driven inflammatory signaling	Ref. [59]
	TLR4 signaling	Microbial LPS activates TLR4/NF-κB signaling	Increased inflammatory cytokine production and tumor promotion	Indicates that FMT may correct excessive microbial inflammatory stimulation	Ref. [57]
	NOD1 signaling	Innate microbial sensing regulates inflammatory tumorigenesis	Modulates intestinal immune balance	Supports restoration of host–microbiota immune equilibrium	Ref. [58]
T-cell functional restoration	*Roseburia intestinalis*	Butyrate-producing bacteria enhance CD8^+^ T-cell effector programs	Increased IFN-γ^+^, TNF-α^+^ and Granzyme B^+^ CD8^+^ T cells; enhanced anti-PD-1 efficacy	Supports transfer of SCFA-producing functional modules through FMT	Ref. [62]
	Microbial metabolites	SCFAs enhance memory potential of CD8^+^ T cells	Improved T-cell persistence and function	Provides metabolic basis for FMT-mediated immune enhancement	Ref. [47]
	Butyrate	Butyrate enhances CD8^+^ T-cell function through metabolic regulation	Increased cytotoxic immunity	Links microbial metabolite restoration with ICI sensitization	Ref. [76]
	CTLA-4 blockade-associated microbiota	Gut microbiota regulates antitumor immune activation during checkpoint blockade	Enhanced therapeutic response	Establishes causal microbiota–ICI interaction	Ref. [26]
Myeloid immune remodeling	SCFA-producing microbiota	Microbial metabolites influence hematopoiesis and myeloid differentiation	Altered inflammatory immune phenotype	Supports metabolite-mediated myeloid remodeling after FMT	Ref. [68]
	MDSC regulation	Regulation of MDSC recruitment and suppressive activity	Reduced T-cell inhibition	Provides rationale for targeting myeloid suppression through microbiota modulation	Refs. [64,65]
	Tumor-associated macrophages	Microenvironmental signals regulate macrophage polarization	Altered M1/M2 balance	Suggests FMT may reshape TAM functional states	Refs. [71,81]
	Gut mycobiota imbalance	Fungal microbiota influences immune suppression	Increased immunosuppressive myeloid cells	Highlights the importance of whole microbial ecosystem remodeling	Ref. [69]
Microbiota-derived metabolic regulation	SCFAs	HDAC inhibition and metabolic reprogramming	Enhanced CTL activity	Supports SCFA restoration as a mechanism of FMT efficacy	Ref. [75]
	Microbial inosine	Adenosine A2A receptor activation	Enhanced checkpoint blockade response	Demonstrates metabolite-mediated immune regulation	Ref. [78]
	Tryptophan metabolites	AhR signaling regulation	Altered immune differentiation	Expands FMT mechanisms beyond SCFAs	Ref. [79]
	Secondary bile acids	FXR/TGR5-mediated regulation	Modulation of Th17/Treg balance	Indicates diverse metabolic outputs of microbiota	Ref. [80]
Tumor-associated microbiota functional plasticity	*Fn*	FadA-mediated E-cadherin/β-catenin activation	Promotes tumor proliferation	Represents classical tumor-promoting microbial activity	Ref. [83]
	*Fn*	Fap2-TIGIT interaction	Suppression of NK/T-cell activity	Demonstrates microbial immune escape mechanisms	Ref. [84]
	*Fn*	TLR4/NF-κB-mediated macrophage remodeling	Increased immune suppression	Links tumor-associated bacteria with immune escape	Refs. [85,86]
	Fn-derived functional metabolite	Butyrate inhibits HDAC3/8, increases Tbx21 acetylation and reduces PD-1 expression	Restored CD8^+^ T-cell function and enhanced anti-PD-1 response	Demonstrates ecological context-dependent immune effects of “oncogenic” bacteria	Ref. [39]
	*Bacteroides uniformis*-derived polysaccharide	Lag-3–NK cell immune activation	Enhanced immune surveillance	Supports identification of transferable microbial immune modules	Ref. [87]

**Table 2:** Microbial mechanisms involved in colorectal cancer progression and context-dependent modulation of antitumor immunity.

Mechanistic Category	Microbial Factor/Intervention	Molecular Mechanism	Immune Consequence	Implication for FMT-Based CRC Immunotherapy	Representative Study
Tumor-promoting signaling	Fn–FadA	FadA binds E-cadherin, promotes β-catenin release, and activates Wnt/β-catenin signaling and inflammatory gene expression.	Promotes epithelial proliferation and tumorigenic transformation.	Consider tumor-promoting microbial functions when characterizing donor microbiota.	Ref. [83]
Tumor progression	Fn–Annexin A1-associated signaling	Induction of Annexin A1 expression and amplification of Wnt/β-catenin signaling.	Promotes CRC cell proliferation, migration, and invasion.	Supports functional characterization of tumor-associated microbial features.	Ref. [89]
Immune escape	Fn–Fap2	Fap2 interacts with TIGIT.	Suppresses NK-cell and T-cell activation and reduces immune-mediated tumor recognition and elimination.	Consider microbial factors associated with immune suppression when evaluating microbiota-based interventions.	Ref. [84]
Immune checkpoint regulation	Fn	Promotes PD-L1 expression in CRC cells.	Facilitates tumor immune evasion.	Evaluate potential relationships between transferred microbial functions and immune checkpoint regulation.	Ref. [90]
Macrophage remodeling	Fn-associated stimulation	TLR4/NF-κB-associated inflammatory signaling.	Promotes immunosuppressive TAM characteristics and reinforces an immunosuppressive microenvironment.	Assess macrophage phenotypes and inflammatory signaling when investigating FMT-mediated immune remodeling.	Ref. [85]
CD8^+^ T-cell dysfunction	Fn enrichment	The supplied paragraph reports an association with reduced CD8^+^ T-cell activity but does not specify a distinct molecular pathway.	Reduced CD8^+^ T-cell activity and increased immunosuppressive-cell infiltration are associated with Fn enrichment.	Evaluate immune-cell changes alongside microbial composition after microbiota transfer.	Ref. [86]
Metabolite-mediated immunotherapy sensitization	Fn-derived butyrate	In MSS CRC models, inhibits HDAC3/8 in CD8^+^ T cells, increases H3K27 acetylation at the Tbx21 promoter, increases TBX21 expression, and reduces PD-1 expression.	Alleviates CD8^+^ T-cell exhaustion and enhances anti-PD-1 efficacy in MSS CRC models.	Supports investigating transferable microbial metabolic functions as potential determinants of FMT-associated immunotherapy responses.	Ref. [39]
NK-cell-mediated immune surveillance	*Bacteroides uniformis* capsular polysaccharide	Activates a LAG-3-associated NK-cell immune axis.	Enhances immune surveillance and improves immunotherapy responses in the reported study.	Supports investigating transferable microbial components for microbiome-based immunomodulation.	Ref. [87]
Inflammation-associated tumorigenesis	*Bacteroides fragilis* toxin	Toxin production and inflammatory signaling.	May contribute to colorectal tumorigenesis; effects may vary with host and microbial context.	Highlights the importance of considering microbial functional traits and ecological context.	Ref. [91]
Metabolic and inflammatory signaling	*Peptostreptococcus anaerobius*	Modulates host metabolic pathways and inflammatory signaling.	Implicated in CRC development; effects may depend on ecological interactions and the tumor environment.	Supports evaluating microbial interactions and functional context rather than taxonomy alone.	Ref. [20]

Abbreviations: Fn, *Fusobacterium nucleatum*; CRC, colorectal cancer; FMT, fecal microbiota transplantation; ICI, immune checkpoint inhibitor; NK, natural killer; TAM, tumor-associated macrophage; TIGIT, T-cell immunoreceptor with Ig and ITIM domains; PD-1, programmed cell death protein 1; PD-L1, programmed death-ligand 1; MSS, microsatellite-stable.

## Data Availability

No new data were generated or analyzed in this study. Data sharing is not applicable to this article.
